# Greater Disease Severity and Worse Clinical Outcomes in Patients Hospitalised with COVID-19 in Africa

**DOI:** 10.5334/gh.1314

**Published:** 2024-04-16

**Authors:** Lina Hahnle, Mathilda Mennen, Freedom Gumedze, Daniel Mutithu, Marguerite Adriaanse, Daniel Egan, Simthandile Mazondwa, Rochelle Walters, Lambert Tetteh Appiah, Francisca Inofomoh, Okechukwu Ogah, Olukemi Adekanmbi, Fastone Goma, Elijah Ogola, Kieran Mwazo, Ahmed Suliman, Kavita Singh, Lana Raspail, Dorairaj Prabhakaran, Pablo Perel, Karen Sliwa, Ntobeko A. B. Ntusi

**Affiliations:** 1 Department of Medicine, University of Cape Town, South Africa; 2 UCT/SAMRC Extramural Unit on Intersection of Noncommunicable Diseases and Infectious Diseases, South Africa; 3 ARUA/GUILD Cluster of Research Excellence on Noncommunicable Diseases and associated multimorbidities, South Africa; 4 Department of Statistical Sciences, University of Cape Town, South Africa; 5 Cape Heart Institute, University of Cape Town, South Africa; 6 Department of Medicine, Kwame Nkrumah University of Science & Technology (KNUST) and Komfo Anokye Teaching Hospital, Kumasi, Ghana; 7 Internal Medicine Department, Olabisi Onabanjo University Teaching Hospital, Nigeria; 8 Department of Medicine, College of Medicine, University of Ibadan, and University College Hospital Ibadan, Nigeria; 9 Department of Medicine, University College Hospital Ibadan, Nigeria; 10 Centre for Primary Care Research, Levy Mwanawasa University Teaching Hospital, Lusaka, Zambia; 11 The Mombasa Hospital, Kenya; 12 Department of Medicine, Coast General Teaching and Referral Hospital, Mombasa, Kenya; 13 Faculty of Medicine, University of Khartoum, Sudan; 14 Public Health Foundation of India, Gurugram, Haryana and Centre for Chronic Disease Control, New Delhi, IN; 15 Heidelberg Institute of Global Health, University of Heidelberg, Germany; 16 World Heart Federation, Geneva, CH; 17 Public Health Foundation India, Centre for Chronic Disease Control, IN; 18 World Heart Federation, CH; 19 London School of Hygiene & Tropical Medicine, GB; 20 Department of Non-communicable Disease Epidemiology, London School of Hygiene & Tropical Medicine, GB; 21 Cape Heart Institute, Department of Medicine & Cardiology, Groote Schuur Hospital, Faculty of Health Sciences, University of Cape Town, South Africa; 22 J46 (J floor) Old Main Building, Groote Schuur Hospital Observatory, 7925, Cape Town, South Africa

**Keywords:** COVID-19, Africa, sub-Saharan Africa, mortality, cardiovascular disease

## Abstract

**Background:**

COVID-19 cardiovascular research from Africa is limited. This study describes cardiovascular risk factors, manifestations, and outcomes of patients hospitalised with COVID-19 in the African region, with an overarching goal to investigate whether important differences exist between African and other populations, which may inform health policies.

**Methods:**

A multinational prospective cohort study was conducted on adults hospitalised with confirmed COVID-19, consecutively admitted to 40 hospitals across 23 countries, 6 of which were African countries. Of the 5,313 participants enrolled globally, 948 were from African sites (n = 9). Data on demographics, pre-existing conditions, clinical outcomes in hospital (major adverse cardiovascular events (MACE), renal failure, neurological events, pulmonary outcomes, and death), 30-day vitality status and re-hospitalization were assessed, comparing African to non-African participants.

**Results:**

Access to specialist care at African sites was significantly lower than the global average (71% vs. 95%), as were ICU admissions (19.4% vs. 34.0%) and COVID-19 vaccination rates (0.6% vs. 7.4%). The African cohort was slightly younger than the non-African cohort (55.0 vs. 57.5 years), with higher rates of hypertension (48.8% vs. 46.9%), HIV (5.9% vs. 0.3%), and Tuberculosis (3.6% vs. 0.3%). In African sites, a higher proportion of patients suffered cardiac arrest (7.5% vs. 5.1%) and acute kidney injury (12.7% vs. 7.2%), with acute kidney injury (AKI) appearing to be one of the strongest predictors of MACE and death in African populations compared to other populations. The overall mortality rate was significantly higher among African participants (18.2% vs. 14.2%).

**Conclusions:**

Overall, hospitalised African patients with COVID-19 had a higher mortality despite a lower mean age, contradicting literature that had previously reported a lower mortality attributed to COVID-19 in Africa. African sites had lower COVID-19 vaccination rates and higher AKI rates, which were positively associated with increased mortality. In conclusion, African patients were hospitalized with more severe COVID-19 cases and had poorer outcomes.

## Introduction

The Coronavirus-19 (COVID-19) pandemic has affected every continent worldwide, impacting the lives of people everywhere. Recent data shows that up to two-thirds of all Africans have been infected with SARS-CoV-2 (the virus which causes COVID-19), which is higher than the global average of 45% [1]. Africa was predicted to be one of the continents to be hardest hit by COVID-19, given the higher burden of Human Immunodeficiency Virus (HIV), Tuberculosis (TB), and Malaria, with the addition of an increasing burden of non-communicable diseases [4]. This, however, was not reflected in follow-up studies showing that deaths due to the virus were low, despite high case rates [2]. Factors such as a younger population, undertesting, and different demographics have been utilized to explain the differences in death rates [2]. This suggests that data regarding COVID-19 should, therefore, be interpreted specifically for the African region. Yet, the amount of research conducted in this area by authors who are affiliated with African research institutions remains low, with only 4.3% of authors being affiliated with Africa [3].

Individuals with pre-existing cardiovascular disease are more prone to severe COVID-19 infections, often requiring intensive care unit (ICU) admission and ventilatory support, and having higher mortality rates [4]. The COVID-19 virus enters a cell through the angiotensin-converting enzyme 2 receptor, which may lead to a systemic proinflammatory state and result in an increase in cardiovascular sequelae such as myocarditis, worsening heart failure, acute myocardial infarction, and sudden cardiac death [5]. A previous study looked at a global patient cohort to investigate the association between cardiovascular risk factors and clinical outcomes in patients with COVID-19 [6]. We evaluated whether differences in demographics, age of population, access to healthcare, and other factors may affect the presentation and outcomes of the COVID-19 disease in patients hospitalised in Africa compared with other regions. Adding to this are differences in the cardiovascular disease burden and the underlying epidemiological transitions within sub-Saharan Africa, including rheumatic heart disease and congenital heart disease, amongst others. Therefore, we sought to investigate the cardiovascular outcomes of patients in sub-Saharan Africa to guide policy and preventive measures.

## Methods

Details on the study rationale, study design, study population, and patient recruitment were reported in Sliwa et al. [7]. In summary, a multinational prospective cohort study was conducted on adults with confirmed COVID-19, consecutively admitted to various hospitals in low-income countries (LICs), low-to-middle income countries (LMICs), upper-middle-income countries (UMICs), and high-income countries (HICs) [8]. Forty hospitals from 23 countries participated in the study, comprising 4 LICs, 15 LMICs, 8 UMICs, and 13 HICs.

### Data collection

Hospital-level data and patient-level data were collected. The latter included information on demographics, clinical characteristics (COVID-19 symptoms and admission vital signs), co-morbidities prior to admission (cardiovascular and non-cardiovascular), pre-admission medications, laboratory tests on admission, other investigations performed during hospitalization (such as Electrocardiogram (ECG), Echocardiogram (ECHO), chest x-ray and Computed Tomography (CT) scan), in-hospital medications, and supportive care during hospital stay. ECG results were uploaded to a web-based platform to be read and codified in a centralized reading centre, according to the Minnesota Code, by experienced and certified cardiologists. Automatic measurements of ECG intervals, including the QT interval, were reviewed [9]. Outcomes data were collected at discharge, including occurrence of major adverse cardiovascular events (MACE), renal failure, neurological events, pulmonary outcomes, and death in the hospital and at 30 days post-discharge. These data were used to determine whether participants were alive, demised (with cause of death noted in this instance), or whether they had required re-admission to hospital.

### Outcomes

Study outcomes included the need for admission to ICU, mechanical ventilation, major adverse cardiovascular events (including cardiac arrest, acute heart failure, atrial fibrillation, myocardial infarction, myocarditis, ventricular arrhythmia, pericarditis, endocarditis, heart blocks, haemorrhagic stroke, ischaemic stroke, and pulmonary embolism), neurological outcomes, acute renal failure, pulmonary outcomes (including pneumonia and acute respiratory distress syndrome), death, and cause of death [7]. Additionally, post-discharge 30-day outcomes included need for readmission to hospital or death post discharge.

### Statistical analyses

For context, we report hospital-level resources by WHO region in percentages. We report demographics, admission vitals, symptoms, pre-existing conditions, laboratory measures, in-hospital outcomes, and 30-day vitality status. Categorical variables are reported as percentages, continuous normally distributed variables as mean (SD), and skewed distributions as median (IQR). Although data are reported by survivor status for each cohort, P-values are reported for overall differences between African and non-African participants. The p-values were assessed using the two-sample t-tests, or Mann-Whitney tests for continuous variables, and the chi-square test or Fisher’s exact test for categorical variables.

The associations between demographic characteristics, clinical characteristics, mortality (in-hospital and overall), and MACE were assessed using logistic regression. Unadjusted and adjusted odd ratios with 95% confidence intervals were reported. In the adjusted models, each of the reported odds ratios were adjusted for age, sex, body mass index (BMI), smoking, vaccine status, diabetes, hypertension, COPD, asthma, HIV, and acute renal injury. All analyses were performed using R Studio version 1.4.1106.

### Ethical considerations

The University of Cape Town granted institutional ethics approval for the study, as well as the coordinating centres in India (PHFI and Centre for Chronic Disease Control, New Delhi) [7]. Investigators from all the participating sites obtained ethics approval from the ethics committees at their respective institutions. National regulatory clearances were obtained, and patients gave informed consent to participate voluntarily in the study.

## Results

**Figure 1:**
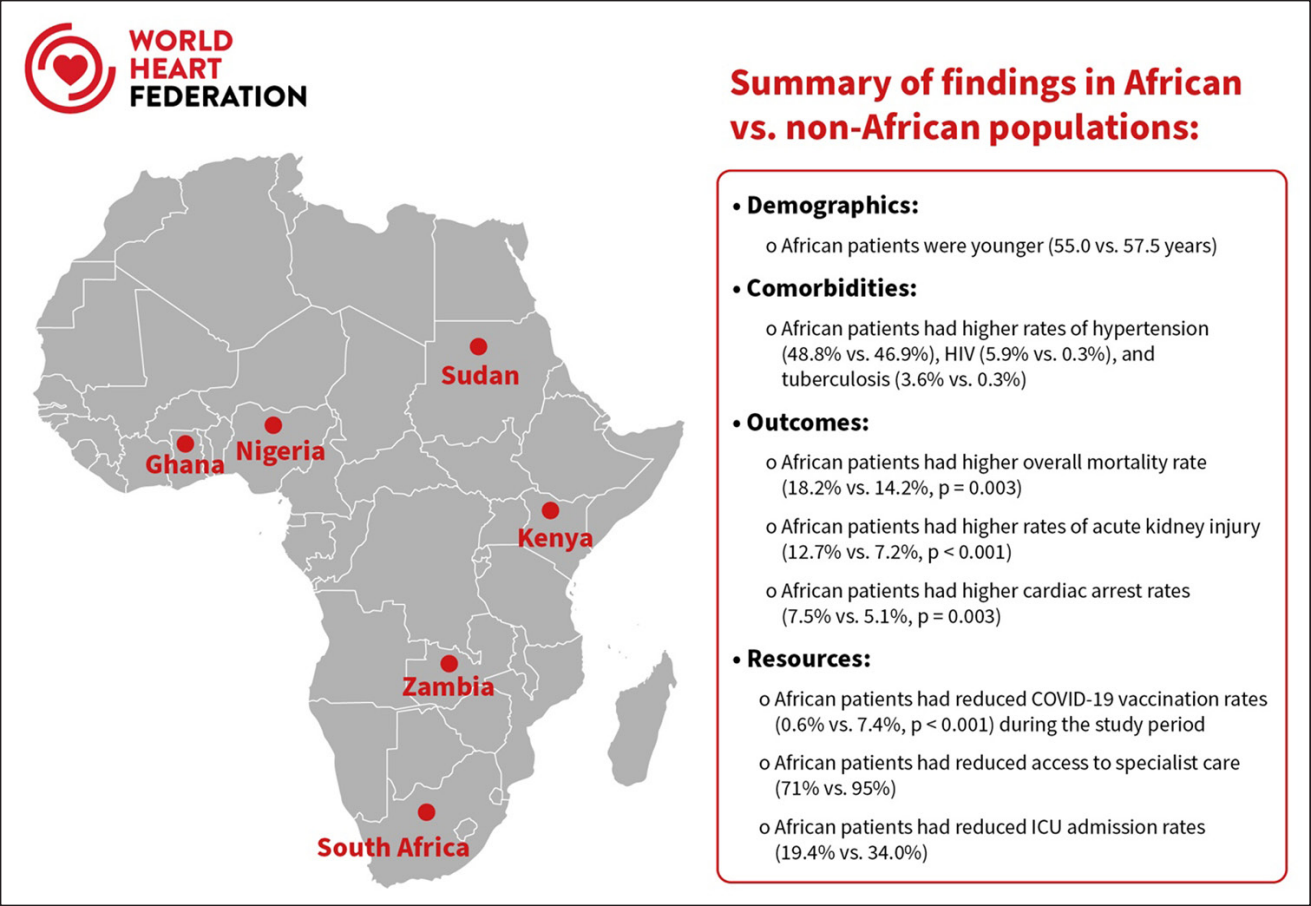
Central figure displaying participating sites and key study findings.

**Figure 2:**
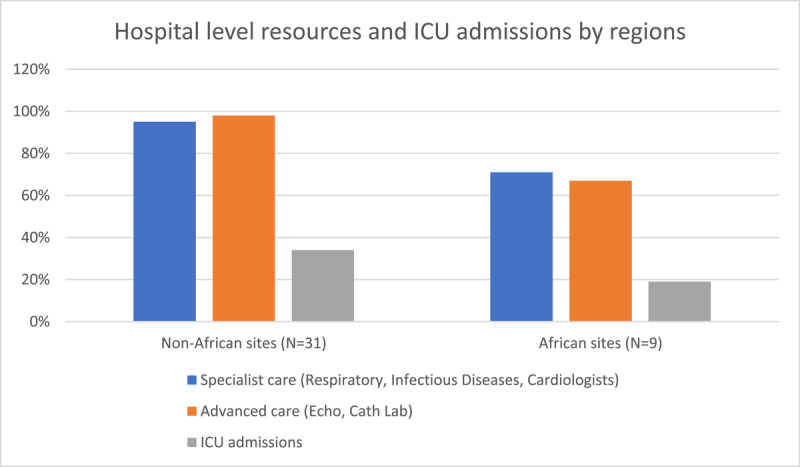
**Hospital level resources and ICU admissions by regions.** Figure 1 shows that sites in the African region had significantly lower rates of specialist care, advanced care, and ICU admissions compared to non-African sites.

Of the 40 recruiting centres, 9 sites were in Africa (LICs = 4, LMICs = 5). In addition to key study findings, Figure 1 displays participating African countries, which were South Africa (n = 189), Zambia (n = 200), Kenya (n = 151), Nigeria (n = 304), Ghana (n = 79), and Sudan (n = 25). African sites had suboptimal specialist services and advanced care compared to higher income countries (Figure 2).

Of the 5,313 COVID-19 patients enrolled across the 40 sites, 948 (17.8%) were from African sites. Table 1 compares the demographic characteristics of the African and non-African study participants. The African cohort had a significantly younger overall mean age of 55.0 years, compared to 57.5 years in the non-African cohort. The sex distribution was similar in both cohorts, but the proportion of Caucasians in the African cohort was significantly lower than in the non-African cohort (0.5% vs. 18.2%), while the percentage of black participants was much higher (78.4% vs. 1.2%). Mean BMI was very similar across groups.

**Table 1:** **Demographic and clinical characteristics of African study participants vs. non-African study participants.** The African cohort was significantly younger and had a significantly higher percentage of black participants compared to the non-African cohort. African sites fell into lower and lower-middle income categories, whereas the non-African cohort was comprised of lower-middle- and higher-income sites. African participants had significantly higher heart rates and rates of hypertension with higher baseline systolic and diastolic blood pressures. Covid-19 vaccination rates were significantly lower in the African cohort.


		AFRICA COHORT			NON-AFRICAN COHORT			P-VALUES FOR DIFFERENCE BETWEEN OVERALL AFRICAN AND NON-AFRICAN VALUES
	
		OVERALL (N = 948)	SURVIVORS (N = 775)	NON-SURVIVORS (N = 173)	OVERALL (4365)	SURVIVORS (3737)	NON-SURVIVORS (N = 628)	

Age	mean (SD)	55.0 (16.0)	53.5 (15.5)	62.1 (16.1)	57.5 (16.0)	56.1 (16.0)	65.6 (13.4)	<0.001

Sex	Female	394 (41.6)	319 (41.2)	75 (43.3)	1760 (40.3)	1551 (41.5)	209 (33.3)	0.5

	Male	554 (58.4)	456 (58.8)	98 (56.6)	2605 (59.7)	2186 (58.5)	419 (66.7)	

Ethnic Origin	Caucasian	5 (0.5)	5 (0.6)	0 (0.0)	795 (18.2)	744 (19.9)	51 (8.1)	<0.001

	Hispanic	11 (1.2)	10 (1.3)	1 (0.6)	531 (12.1)	393 (10.5)	138 (21.9)	

	Black	743 (78.4)	620 (80.0)	123 (71.1)	53 (1.2)	49 (1.3)	4 (0.6)	

	Middle Eastern	6 (0.6)	5 (0.6)	1 (0.6)	309 (7.0)	278 (7.4)	31 (4.9)	

	Asian	22 (22.3)	16 (2.1)	6 (3.5)	2420 (55.4)	2030 (54.3)	390 (62.1)	

	Other	159 (16.8)	117 (15.1)	42 (24.3)	200 (4.5)	186 (4.9)	14 (2.2)	

	Unknown	2 (0.2)	2 (0.3)	0 (0.0)	57 (1.3)	57 (1.5)	0	

World Bank Income Groups	LIC	376 (39.7)	331 (42.7)	45 (26.0)	0	0	0	<0.001

	LMIC	383 (40.4)	303 (39.1)	80 (46.2)	2250 (51.5)	1838 (49.2)	412 (65.6)	

	MIC	189 (19.9)	141 (18.2)	48 (27.7)	748 (17.1)	601 (16.1)	147 (23.4)	

	HIC	0	0	0	1367 (31.3)	1298 (34.7)	69(11.0)	

Body Mass Index	(Kg/m^2^), mean (SD)	26.9 (7.4)	26.6 (7.55)	28.8 (6.3)	26.8 (5.2)	26.9 (5.18)	26.5 (5.09)	<0.001

	Underweight (<18)	38 (4.0)	29 (3.7)	10 (5.8)	45 (1.0)	38 (1.0)	7 (1.1)	

	Normal weight (18–24.99)	115 (12.1)	105 (13.5)	1 (0.6)	1302 (29.8)	1140 (30.5)	162 (25.7)	

	Overweight (25–29.99)	113 (13.8)	101 (14.8)	12 (6.9)	1168 (26.8)	1032 (27.6)	136 (21.6)	

	Obese (≥ 30)	131 (13.8)	115 (14.8)	16 (9.2)	697 (16.0)	615 (16.4)	82 (13.0)	

	Unknown	551 (58.1)	425 (54.8)	134 (77.5)	1153 (26.4)	912 (24.4)	241 (38.3)	

Cough		609 (64.0)	494 (63.7)	115 (66.4)	3015 (69.1)	2593 (69.3)	422 (67.2)	0.005

Dyspnoea or Tachypnoea		580 (61.2)	435 (56.1)	145 (83.8)	2728 (62.5)	2254 (60.3)	474 (75.5)	0.5

Heart rate (beats/min), mean (SD)		94.3 (19.8)	92.6 (18.3)	102 (24.1)	91.5 (17.2)	90.9 (16.7)	95.3 (19.8)	<0.001

Systolic BP (mmHg), mean (SD)		133.7 (22.6)	134.1 (21.2)	132 (28.0)	128.0 (20.3)	128.0 (19.5)	129.0 (24.7)	<0.001

Diastolic BP (mmHg), mean (SD)		81.4 (14.7)	82.3 (13.9)	77.1 (17.1)	77.5 (12.5)	77.7 (12.1)	76.4 (14.8)	<0.001

Comorbidities (cardiovascular)	Hypertension	463 (48.8)	356 (45.9)	107 (61.8)	2048 (46.9)	1704 (45.6)	344 (54.8)	0.09

	Diabetes	240 (25.3)	172 (22.2)	68 (39.3)	1460 (33.4)	1174 (31.4)	286 (45.5)	<0.001

	Coronary Artery disease	35 (3.7)	26 (3.4)	9 (5.2)	545 (12.4)	420 (11.2)	125 (19.9)	<0.001

	Heart Failure	37 (3.9)	32 (4.1)	5 (2.9)	253 (5.8)	206 (5.5)	47 (7.4)	0.02

	Stroke	35 (3.7)	29 (3.7)	6 (3.4)	162 (3.7)	130 (3.4)	32 (5.0)	0.6

	Atrial Fibrillation	11 (1.2)	7 (0.9)	4 (2.3)	148 (3.4)	127 (3.4)	21 (3.3)	<0.001

	Peripheral Vascular Disease	12 (1.3)	9 (1.2)	3 (1.7)	94 (2.2)	76 (2.0)	18 (2.8)	0.1

	Cardiomyopathies	13 (1.4)	10 (1.3)	3 (1.7)	47 (1.1)	43 (1.1)	4 (0.6)	0.5

	Rheumatic Heart Disease	2 (0.2)	2 (0.3)	0	54 (1.2)	47 (1.3)	7 (1.1)	0.003

	Chagas Disease	0	0	0	36 (0.8)	34 (0.9)	2 (0.3)	<0.001

	Congenital Heart disease	3 (0.3)	3 (0.4)	0	179 (4.1)	163 (4.3)	16 (2.5)	<0.001

	Valvular Disease	8 (0.8)	6 (0.8)	2 (1.2)	110 (2.5)	88 (2.4)	22 (3.5)	<0.001

Comorbidities (non-cardiovascular)	Chronic Kidney Disease	54 (5.7)	34 (4.4)	20 (11.5)	350 (8.0)	265 (7.1)	85 (13.5)	<0.001

	Chronic Pulmonary disease	20 (2.1)	15 (1.9)	5 (2.9)	189 (4.3)	145 (3.8)	44 (7.0)	0.002

	Asthma	38 (4.0)	33 (4.3)	5 (2.9)	181 (4.1)	167 (4.4)	14 (2.2)	0.2

	Chronic Immunosuppression	24 (2.5)	18 (2.3)	6 (3.4)	112 (2.6)	92 (2.4)	20 (3.1)	<0.001

	HIV	56 (5.9)	48 (6.2)	8 (4.6)	15 (0.3)	14 (0.4)	1 (0.1)	<0.001

	Tuberculosis	34 (3.6)	31 (4.0)	3 (3.4)	22 (0.3)	18 (0.5)	4 (0.6)	<0.001

	Cancer on Chemotherapy	6 (0.6)	3 (0.4)	3 (3.4)	108 (2.5)	87 (2.3)	21 (3.3)	0.003

	Renal Replacement Therapy	6 (0.6)	3 (0.4)	3 (3.4)	56 (1.3)	42 (1.1)	14 (2.2)	<0.001

	Previous Organ Transplant	2 (0.2)	1 (0.1)	1 (0.5)	43 (1.0)	37 (0.9)	6 (0.9)	<0.001

	Covid Vaccine	6 (0.6)	5 (0.6)	1 (0.5)	325 (7.4)	323 (8.6)	2 (0.3)	<0.001


Table 1 also compares the symptoms and clinical signs between the two cohorts. Presentation with self-reported coughing and tachypnea were similar between the two groups. The African cohort had a significantly higher mean heart rate (94.7 vs. 91.5), and significantly higher systolic (133.7 vs. 128), as well as diastolic (81.3 vs. 77.5) pressures, compared to non-African participants. The African cohort had a higher prevalence of hypertension (48.8% vs. 46.9%), HIV (5.9% vs. 0.3%), and TB (3.6% vs. 0.3%), but a significantly lower rates of diabetes (25.3% vs. 33.4%) and a much lower rate of COVID-19 vaccination rate (0.6% vs. 7.4%).

Table 2 shows laboratory findings between the two cohorts. African participants had a higher WCC and a significantly higher CRP (75.7 vs. 51) than non-African participants. No differences were seen in creatinine, D-dimers, or HbA1c. The African non-survivors seemed to have higher pro-BNP levels (163.7 vs. 130.4), total cholesterol levels (4.3 vs. 3.4), and LDL cholesterol levels (3.3 vs. 1.6), as compared to non-African non-survivors. T wave changes were more common (17.9% vs. 13.8%) and QTc longer (437 ms vs. 413 ms) in the African participants on their admission ECGs. ICU admissions were fewer (19.4 vs. 34.0%) in the African cohort. However, participants in the African cohort had higher rates of cardiac arrest (7.5% vs. 5.1%). Other major adverse events were comparable between the two groups. Rates of pneumonia and ARDS also showed no statistically significant differences. Acute renal injury was more common in African participants (12.7% vs. 7.2%).

**Table 2:** **In-hospital findings of participants with Covid-19.** African participants had a higher WCC and a significantly higher CRP compared to non-African participants. African non-survivors had higher pro-BNP levels, higher total cholesterol levels, and LDL cholesterol levels compared to non-African non-survivors. T wave changes were more common and QTc longer in the African participants on their admission ECGs. ICU admissions rates were lower in the African cohort, but with higher rates of cardiac arrest. Other major adverse events were comparable between the two groups. Rates of pneumonia and ARDS also showed no statistically significant differences. Acute renal injury was more common in African participants.


		AFRICA COHORT			NON-AFRICAN COHORT			P-VALUES FOR DIFFERENCE BETWEEN OVERALL AFRICAN AND NON-AFRICAN VALUES
	
		OVERALL (N = 948)	SURVIVORS (N = 775)	NON-SURVIVORS (N = 173)	OVERALL (N = 4365)	SURVIVORS (3737)	NON-SURVIVORS (N = 628)	

**Laboratory findings (median; IQR)**	WCC, ×10^9/L	2.0 (0.00, 8.8)	2.6 (0.0, 8.5)	0.03 (0.01, 9.3)	4.8 (0.0, 8.3)	5.2 (0.0, 8.5)	0.02 (0.0, 6.9)	0.3

	Hb, mml/L	7.9 (6.9, 8.7)	8.0 (7.1; 8.8)	7.6 (6.4; 8.6)	8.0 (7.1; 8.8)	8.0 (7.2; 8.8)	7.8 (6.6; 8.7)	0.02

	Platelets, 10^3/uL	238 (180; 315)	239 (182; 317)	230 (174; 308)	230 (166; 343)	231 (168; 347)	216 (152; 318)	<0.001

	CRP, mg/L	75.7 (24.5; 142.5)	68.1 (20.9; 133)	105.4 (52.3; 197.8)	51 (16.6; 103.6)	46.3 (15.1; 99.3)	91 (34.3; 163.4)	<0.001

	Creatinine, umol/L	84.4 (69; 114.9)	82 (67.9; 106.3)	104.4 (74.3; 144.8)	88.0 (70.7; 113.2)	85.8 (69.8; 107)	99.5 (76.3; 155.7)	0.2

	D-Dimer, mg/FEU/L	1.0 (0.4; 5.9)	0.8 (0.3; 6.1)	1.9 (0.5: 5.2)	1.0 (0.5; 4:0)	0.9 (0.4; 3.3)	2.0 (0.8; 5.4)	0.7

	NT-Pro-BNP, pmol/L	60.5 (15; 257.1)	19.6 (7.1; 85.2)	163.7 (37.6; 480.7)	60.1 (12; 254.0)	47.4 (10.3; 226.1)	130.4 (41.4; 454.0)	0.2

	HbA1c	6.8 (6.1; 8.6)	6.8 (6.1; 8.7)	6.9 (6.3; 8.6)	6.9 (6.1, 8.4)	6.9 (6.1; 8.5)	6.9 (6.2; 8.4)	0.3

	IL-6, pg/ml	-	-	-	25.2 (8.7; 64.7)	21.6 (7.0; 52.0)	64.5 (21.3; 125.0)	-

	Total cholesterol. mmol/L	4.2 (3.3; 5.1)	4.2 (3.3; 5.2)	4.3 (2.9; 4.9)	4.0 (3.1; 5.0)	4.2 (3.4; 5.2)	3.4 (2.7; 4.3)	0.6

	HDL	1.0 (0.8; 1.2)	1.0 (0.8; 1.2)	1.0 (0.8; 1.1)	0.9 (0.8; 1.1)	1.0 (0.8; 1.1)	0.8 (0.6; 1.1)	0.4

	LDL	2.5 (1.6, 3.5)	2.5 (1.8; 3.6)	3.3 (1.1; 3.3)	2.2 (1.6; 2.9)	2.3 (1.8; 3.0)	1.6 (1.2; 2.2)	0.8

	Triglycerides	1.5 (1.0; 2.1)	1.5 (1.1; 2.2)	1.1 (1.0; 1.5)	1.6 (1.2; 2.1)	1.6 (1.2; 2.1)	1.6 (1.3; 2.3)	0.2

**ECG Findings**	T wave changes (%)	170 (17.9)	131 (16.9)	39 (22.5)	605 (13.8)	463 (12.3)	142 (22.6)	0.005

	AF (%)	21 (2.2)	13 (1.7)	8 (4.6)	110 (2.5)	84 (2.2)	26 (4.1)	<0.001

	QTc (median, IQR)	437 (406; 460)	433 (403; 459)	447 (420; 472)	413 (1; 443)	410 (1; 440)	424 (352; 456)	<0.001

**Median Length of Hospital Stay in days (IQR)**		9 (6, 13)	9 (6, 13)	10 (5.25, 11.50)	10 (6,15)	10 (6,15)	11 (6, 17)	0.3

**ICU admission (%)**		184 (19.4)	110 (14.2)	74 (42.7)	1484 (34.0)	1063 (28.4)	421 (67.0)	<0.001

**Major Adverse Cardiovascular Events (%)**	Cardiac Arrest	71 (7.5)	0	71 (41.0)	223 (5.1)	56 (1.4)	167 (26.6)	0.003

	Acute heart Failure	35 (3.7)	23 (3.0)	12 (6.9)	166 (3.8)	78 (2.1)	88 (14.0)	0.2

	Atrial Fibrillation	15 (1.6)	1 (0.1)	9 (5.2)	111 (2.5)	80 (2.1)	31 (4.9)	0.1

	Myocardial Infarction	8 (0.8)	4 (0.5)	4 (2.3)	76 (1.7)	59 (1.5)	17 (2.7)	0.003

	Myocarditis	9 (0.9)	5 (0.6)	4 (2.3)	38 (0.8)	17 (0.4)	21 (3.3)	0.03

	Ventricular Arrythmia	8 (0.8)	3 (0.4)	5 (2.8)	52 (1.1)	35 (0.9)	17 (2.7)	0.4

	Pericarditis	13 (1.4)	12 (1.5)	1 (0.5)	17 (0.3)	15 (0.4)	2 (0.3)	<0.001

	Endocarditis	1 (0.1)	1 (0.1)	0 (0.0)	8 (0.1)	5 (0.1)	3 (0.4)	0.5

	Heart Blocks	9 (0.9)	7 (0.9)	2 (1.2)	68 (1.5)	51 (1.3)	17 (2.7)	0.3

	Haemorrhagic Stroke	0 (0.0)	0 (0.0)	0 (0.0	21 (0.4)	14 (0.3)	7 (0.1)	0.07

	Ischaemic Stroke	6 (0.6)	3 (0.4)	3 (1.7)	50 (1.1)	35 (0.9)	15 (2.3)	0.3

	Pulmonary Embolus	11 (1.2)	8 (1.0)	3 (1.7)	105 (2.4)	79 (1.9)	26 (4.1)	<0.001

**Respiratory Outcomes**	Pneumonia	338 (35.7)	252 (32.5)	86 (49.7)	1654 (37.8)	1324 (35.4)	330 (52.5)	0.2

	Acute Respiratory Distress Syndrome	116 (12.2)	58 (7.5)	58 (33.5)	607 (13.9)	363 (9.7)	244 (38.9)	<0.001

**Acute Renal Injury**		120 (12.7)	63 (8.1)	57 (32.9)	316 (7.2)	144 (3.8)	172 (27.3)	<0.001

**Other**	Anaemia	87 (9.2)	58 (7.5)	29 (16.7)	359 (8.2)	253 (6.7)	106 (16.8)	0.4

	Shock	37 (3.9)	4 (0.5)	33 (19.0)	251 (5.7)	48 (1.2)	203 (32.3)	0.05

	Liver Dysfunction	43 (4.5)	30 (3.9)	13 (7.5)	192 (4.3)	142 (3.7)	50 (0.8)	<0.001


Table 3 shows in-hospital outcomes, with death occurring in 17% of admitted African participants, compared to 11.9% in the non-African cohort (p < 0.05). Although 30-day mortality was less in the African cohort, overall mortality was much higher in the African cohort (18.2% vs. 14.2%, p < 0.05).

**Table 3:** **In-hospital outcomes, outcomes at 30 days, and overall causes of death in African and non-African participants.** In-hospital death was more common in hospitalised African participants compared to non-African participants. Although 30-day mortality was lower in the African cohort, overall mortality was significantly higher in the African cohort.


	AFRICAN (N = 948)	NON-AFRICAN (N = 4365)	P-VALUE FOR DIFFERENCE

**In-hospital outcomes (%)**			0.03

Discharged	682 (71.9)	3391 (77.7)	

Alive, and still an inpatient	7 (0.7)	157 (3.6)	

Transfer to another facility	73 (7.7)	155 (3.6)	

Death	**162 (17.0)**	**521 (11.9)**	

Palliative discharge	0 (0.0)	3 (0.1)	

Unknown	24 (2.5)	138 (3.2)	

**Outcomes at 30 days (%)**			<0.001

Alive	659 (69.5)	2968 (68.0)	

Re-hospitalized	5 (0.5)	67 (1.5)	

Death	11 (1.2)	107 (2.5)	

Unknown/loss-to-follow-up	79 (8.3)	589 ((13.5)	

**CAUSE OF DEATH (IN-HOSPITAL + 30-DAY MORTALITY)**	**(N = 173)**	**(N = 628)**	**<0.001**

Sudden Cardiac Death	30 (17.3)	124 (19.7)	

Presumed Cardiovascular	21 (12.1)	20 (3.2)	

Death due to MI	3 (1.7)	5 (0.8)	

Respiratory Failure	69 (39.9)	249 (39.6)	

Death due to Heart Failure	3 (1.7)	46 (7.3)	

Pulmonary embolus	2 (1.2)	8 (1.3)	

Death due to Stroke	0 (0.0)	31 (4.9)	

Other	41 (23.7)	134 (21.3)	

Unknown	4 (2.3)	11 (1.8)	

**Overall mortality**	**173 (18.2)**	**628 (14.2)**	0.003


Tables 4a and 4b show results from crude and adjusted regression analyses for factors associated with in-hospital deaths and overall mortality in patients with COVID-19. Adjusted models accounted for age, sex, BMI, smoking, COVID-19 vaccine status, diabetes, hypertension, COPD, asthma, HIV, and acute renal injury.

**Table 4a:** **Logistic regression analyses for factors associated with in-hospital death in COVID-19 hospitalized patients** – ***Adjusted for age, sex, BMI, smoking, vaccine status, diabetes, hypertension, COPD, asthma, HIV, and acute renal injury*.** Both the African and non-African cohorts showed significantly increased odds of death in participants aged 60 or older. The non-African cohort had higher odds of overall mortality in participants aged 45–60, compared to individuals 45 or younger. Being female was associated with lower odds of overall mortality in the non-African cohort compared to being male. This sex difference was not observed in the African cohort in overall mortality. In the African cohort, smoking status did not significantly change the odds of in-hospital death, whereas in the non-African cohort, smoking was protective. Vaccination status was associated with a strong protective status in the non-African cohort. Diabetes showed a significantly higher risk for in-hospital deaths both in the African and non-African cohorts. Acute renal injury showed the highest increase in odds for in-hospital death, especially in the African cohort.


		AFRICAN COHORT		NON-AFRICAN COHORT	
	
		UNADJUSTED ODDS OR (95% CI)	ADJUSTED ODDS* OR (95% CI)	UNADJUSTED ODDS OR (95% CI)	ADJUSTED ODDS* OR (95% CI)

**Demographic**					

**Age**	**<45 (Ref)**	1.0	1,0	1.0	1.0

	**45–60**	1.09 (0.64, 1.87)	0.63 (0.14, 2.81)	2.63 (1.85, 3.80)	1.91 (1.25, 3.00)

	**>60**	3.28 (2.11, 5,23)	2.95 (0.86, 11.33)	4.90 (3.56, 6.90)	3.73 (2.52, 5.67)

**Sex**	**Male**	1.0	1,0	1.0	1.0

	**Female**	1.12 (0.79, 1.57)	1.81 (0.67, 5.08)	0.76 (0.63, 0.92)	0.76 (0.58, 1.00)

**History**					

**Smoking status**	**Never (Ref)**	1.0	1.0	1.0	1.0

	**Current**	1.96 (0.71, 4.67)	10.57 (0.46, 101.00)	0.36 (0.21, 0.59)	0.44 (0.24, 0.76)

	**Former**	2.15 (1.04, 4.18)	1.54 (0.22, 8.33)	0.92 (0.70, 1.19)	0.64 (0.45, 0.90)

**BMI**	**18–24**	1.0	1,0	1.0	1.0

	**<18**	0.32 (0.02, 1.78)	0.27 (0.01, 1.92)	1.05 (0.36, 2.48)	0.88 (0.27, 2.31)

	**25–29**	1.27 (0.50, 3.27)	0.78 (0.23, 2.54)	1.01 (0.78, 1.31)	0.94 (0.71, 1.25)

	**>30**	1.52 (0.65, 3.76)	1.03 (0.32, 3.33)	0.99 (0.73, 1.33)	0.85 (0.60, 1.19)

**COVID vaccine status**	**Unvaccinated**	1.0	1,0	1.0	1.0

	**Vaccinated**	0.39 (0.02, 2.49)	–	0.02 (0.00, 0.08)	0.09 (0.00, 0.40)

**Clinical**					

**Comorbidities**	**DM**	2.47 (1.72, 3.52)	3.51 (1.31, 9.62)	1.82 (1.51, 2.19)	1.43 (1.10, 1.85)

	**COPD**	1.22 (0.35, 3.37)	–	2.06 (1.42, 2.93)	2.03 (1.19, 3,36)

	**Asthma**	0.72 (0.24, 1.72)	1.69 (0.08, 11.47)	0.56 (0.30, 0.95)	0.55 (0.26, 1.07)

	**Hypertension**	1.98 (1.40, 2.83)	1.96 (0.68, 5.96)	1.49 (1.4, 1.79)	0.89 (0.68, 1.16)

	**HIV**	0.57 (0.22, 1.25)	0.74 (0.06, 4.45)	–	–

**In-hospital complications**	**Acute renal injury**	5.69 (3.75, 8.62)	48.12 (13.44, 197.50)	10.36 (8.10, 13.25)	8.27 (6.07, 11.27)


**Table 4b:** Logistic Regression analysis for factors associated with overall mortality in Covid-19 hospitalised patients. *Adjusted for demographic variables age, sex, BMI, smoking, vaccine status, diabetes, hypertension, COPD, asthma, HIV, and acute renal injury. In the non-African cohort, both participants aged >60 and between 45–60 had higher odds of overall mortality. In the African cohort, only those above the age of 60 were at higher risk of mortality. There was no sex difference in the African participants associated with mortality, but in the non-African cohort, being of female sex was protective. Current smoking status appeared protective only in the non-African cohort. Diabetes was associated with a significantly higher mortality in both groups. AKI showed increased mortality in both groups in adjusted models, but showed a much higher risk of death in the African cohort.


		AFRICAN COHORT		NON-AFRICAN COHORT	
	
		UNADJUSTED ODDS OR (95% CI)	ADJUSTED ODDS* OR (95% CI)	UNADJUSTED ODDS OR (95% CI)	ADJUSTED ODDS* OR (95% CI)

**Demographic**					

**Age**	**<45 (Ref)**	1.0	1,0	1.0	1.0

	**45–60**	1.01 (0.60, 1.71)	0.66 (0.15, 2.76)	2.76 (2.00, 3.89)	2.14 (1.42, 3.29)

	**>60**	3.42 (2.23, 5.38)	4.03 (1.26, 14.73)	5.27 (3.92, 7.24)	4.18 (2.86, 6.26)

**Sex**	**Male**	1.0	1,0	1.0	1.0

	**Female**	1.09 (0.78, 1.52)	1.95 (0.76, 5.17)	0.70 (0.59, 0.84)	0.71 (0.55, 0.91)

**History**					

**Smoking status**	**Never (Ref)**	1.0	1,0	1.0	1.0

	**Current**	2.00 (0.78, 4.57)	8.04 (0.36, 72.45)	0.43 (0.27, 0.66)	0.53 (0.31, 0.86)

	**Former**	1.82 (0.88, 3.50)	1.15 (0.17, 5.86)	0.98 (0.76, 1.25)	0.70 (0.50, 0.97)

**BMI**	**18–24**	1.0	1,0	1.0	1.0

	**<18**	0.28 (0.02, 1.56)	0.24 (0.01, 1.64)	1.30 (0.52, 2.78)	1.19 (0.43, 2.85)

	**25–29**	1.25 (0.52, 3.08)	0.77 (0.25, 2,36)	0.93 (0.73,1.18)	0.85 (0.65, 1.11)

	**>30**	1.46 (0.64, 3.47)	0.98 (0.33, 2.96)	0.94 (0.70, 1.24)	0.81 (0.59, 1.11)

**COVID vaccine status**	**Unvaccinated**	1.0	1,0	1.0	1.0

	**Vaccinated**	0.37 (0.02, 2.33)	–	0.03 (0.0, 0.09)	–

**Clinical**					

**Comorbidities**	**DM**	2.31 (1.62, 3.28)	3.07 (1.20, 7.92)	1.81 (1.52, 2.14)	1.43 (1.11, 1.83)

	**COPD**	1.51 (0.48, 3.95)	–	1.86 (1.30, 2.61)	2.05 (1.24, 3.31)

	**Asthma**	0.67 (0.23, 1.59)	1.11 (0.06, 7.29)	0.49 (0.27, 0.81)	0.47 (0.22, 0.90)

	**Hypertension**	1.94 (1.39, 2.74)	1.82 (0.67, 5.17)	1.39 (1.17, 1.65)	0.91 (0.70, 1.17)

	**HIV**	0.74 (0.32, 1.50)	0.63 (0.06, 3.43)	0.42 (0.02, 2.11)	–

**In-hospital complications**	**AKI**	5.82 (3.85, 8.79)	41.74 (12.02, 164.04)	9.16 (7.19, 11.68)	8.02 (5.91, 10.90)


In the non-African cohort, participants over the age of 60 showed 3.73 times higher odds of in-hospital death, compared to those younger than 45. The increased odds in the African cohort were not statistically significant. However, regarding overall mortality, both the African and non-African cohorts showed significantly increased odds of death in participants aged 60 or older (OR of 4.03 vs. 4.18 respectively). Interestingly, the non-African cohort showed higher odds of overall mortality, even in participants aged 45–60 compared to individuals younger than 45 years of age.

Being female was associated with lower odds of overall mortality (0.71) in the non-African cohort, as compared to being male. This sex difference was not observed in the African cohort in overall mortality. Vaccination status was associated with a strong protective status in the non-African cohort. It could not, however, be elicited in the African cohort due to very low vaccination rates and poor reporting. This was seen both in the in-hospital deaths and overall mortality analyses.

Diabetes showed a significantly higher risk for in-hospital deaths in both the African and non-African cohorts, but particularly in the African cohort, with adjusted ORs of 3.51 and 1.43, respectively. Regarding overall mortality, the adjusted odds of death were still increased for those with diabetes, although they were slightly less than for in-hospital death. Hypertension was associated with higher odds of death, but only in the unadjusted analyses for both in-hospital and overall mortality. When adjusting for covariates, no significant increase in odds was found. HIV was not associated with a higher risk of in-hospital death or overall mortality. AKI showed the highest increase in odds for in-hospital death, with an odds ratio of 48.12 in the African cohort and 8.27 in the non-African cohort, and similar results for overall mortality.

Table 4c shows the results from regression analyses assessing risk factors associated with major adverse cardiac events (MACE). Age above 60 was associated with higher odds of MACE in the non-African cohort, being 2.82 times higher in the adjusted model. However, no difference in the odds of MACE occurrence was found across age groups in the African cohort. Being female was protective in the non-African cohort, but females in the African cohort had 19.95 times the odds of developing MACE, compared to their male counterparts. Covid vaccination status again showed lower odds of developing MACE in the non-African cohort, but it could not be reported in the African participants. Diabetes was associated with higher odds of developing MACE in the non-African cohort (1.32), but not in the African cohort. Again, acute renal injury was one of the strongest predictors of adverse cardiac outcomes in both cohorts, especially in the African cohort where AKI was associated with 660.55 higher odds of MACE.

**Table 4c:** **Logistic Regression analysis for factors associated with MACE. *Adjusted for demographic variables age, sex, BMI, smoking, vaccine status, diabetes, hypertension, COPD, asthma, HIV, and acute renal injury*.** In adjusted models, age >60 was associated with MACE only in the non-African cohort. Among African participants, being female was significantly associated with occurrence of MACE, whereas in the non-African cohort, being female was protective. Covid vaccination status appeared to have no impact on MACE. Diabetes was associated with a higher odds of MACE occurrence in the non-African cohort, but not in the African one. Again, acute renal injury appeared to be one of the strongest predictors of adverse cardiac outcomes in both cohorts.


		AFRICAN COHORT		NON-AFRICAN COHORT	
	
		UNADJUSTED ODDS OR (95% CI)	ADJUSTED ODDS* OR (95% CI)	UNADJUSTED ODDS OR (95% CI)	ADJUSTED ODDS* OR (95% CI)

**Demographic**					

**Age**	**<45 (Ref)**	1.0	1,0	1.0	1.0

	**45–60**	0.87 (0.50, 1.51)	0.28 (0.01, 5.22)	2.75 (2.06, 3.70)	2.06 (1.48, 2.89)

	**>60**	2.10 (1.34, 3.37)	1.95 (0.18, 30.89)	3.94 (3.02, 5.22)	2.82 (2.06, 3.90)

**Sex**	**Male**	1.0	1,0	1.0	1.0

	**Female**	1.22 (0.84, 1.77)	19.95 (2.65, 440.97)	0.77 (0.65, 0.92)	0.68 (0.54, 0.84)

**History**					

**Smoking status**	**Never (Ref)**	1.0	1,0	1.0	1.0

	**Current**	2.90 (1.23, 6.28)	–	0.51 (0.34, 0.74)	0.48 (0.30, 0.73)

	**Former**	2.09 (1.03, 3.98)	2.48 (0.11, 53.07)	1.31 (1.05, 1.61)	0.95 (0.73, 1.24)

**BMI**	**18–24**	1.0	1,0	1.0	1.0

	**< 18**	–	–	0.92 (0.37, 1.97)	1.04 (0.40, 2.38)

	**25–29**	1.37 (0.30, 7.09)	0.95 (0.06, 16.17)	0.93 (0.75, 1.16)	0.84 (0.66, 1.05)

	**>30**	3.42 (1.04, 15.42)	1.01 (0.09, 14.47)	1.09 (0.86, 1.39)	0.94 (0.72, 1.23)

**COVID vaccine status**	**Unvaccinated**	1.0	1,0	1.0	1.0

	**Vaccinated**	1.16 (0.06, 7.53)	–	0.20 (0.11, 0.35)	0.51 (0.22, 1.02)

**Clinical**					

**Comorbidities**	**DM**	1.90 (1.28, 2.81)	1.35 (0.15, 10.61)	1.65 (1.40, 1.95)	1.32 (1.06, 1.64)

	**COPD**	5.42 (2.14, 13.36)	1.47 (0.00, 401.57)	1.57 (1.09, 2.21)	1.13 (0.69, 1.78)

	**Asthma**	0.73 (0.21, 1.87)	–	1.12 (0.74, 1.64)	0.96 (0.58, 1.52)

	**Hypertension**	1.49 (1.03, 2.18)	1.54 (0.22, 12.14)	1.97 (1.67, 2.34)	1.30 (1.04, 1.63)

	**HIV**	0.60 (0.21, 1.39)	0.08 (0.00, 6.12)	0.38 (0.02, 1.90)	–

**In-hospital complications**	**AKI**	16.62 (10.65, 26.20)	660.55 (75.13, 18871.12)	5.94 (4.68, 7.54)	4.92 (3.67, 6.58)


## Discussion

Our analysis showed important differences comparing the African cohort to the global cohort data. The African cohort had a significantly higher mortality rate and poorer overall access to specialist care.

On the African continent, specialist care was only present in 71% of the hospitals, which was lower than any other continent in the global study. Advanced care, which included echocardiography facilities and catheterization laboratories, was only found in 67% of the hospitals, indicating an under-resourced system. Within the global cohort, the majority of participating sites were of lower-middle or high-income status, whereas the African cohort comprised mostly low or low-middle income groups.

Our findings of higher heart rates on admission, worse prognostic ECG changes, higher rates of AKI, and higher rates of sudden cardiac death among patients hospitalised with COVID-19 in Africa, have noteworthy clinical implications. These findings not only challenge the prevailing notion that COVID-19 was milder among Africans, but also highlight the critical need for healthcare providers and policy planners to prioritize enhancing clinical capacity and critical care resources in regions where patients suffered worse outcomes.

At baseline, the African cohort had slightly higher systolic and diastolic blood pressures, averaging 5 mmHg higher than their non-African counterparts. Prevalence of hypertension has steadily been increasing on the African continent, from 19.7% in 1990 to 30.8% in 2010 [10]. In the African cohort, 48.8% had hypertension as a comorbidity (vs. 46% in the non-African cohort), making it the most common co-morbidity in the African cohort. Hypertension is a major health challenge on the African continent and, while communicable diseases often get a lot of attention, cardiovascular disease is more deadly than cancer, chronic respiratory disease, and diabetes combined. Further, between 2010 and 2019, the African continent had the second highest mortality rate from non-communicable diseases [11,12]. In-hospital cardiac arrest was also higher at 7.5% compared to 5.1% in the non-African cohort. Interestingly, no clinically meaningful differences were observed in BMI between the two cohort, but the non-African cohort had a higher proportion of obesity (26.8% vs. 13.8% in the African cohort).

Mortality overall was significantly higher in the African cohort at 18.2% (compared with 14.2% in the non-African group). The African cohort has lower ICU admission rates, reflecting a system that had less ICU bed availability, potentially contributing to the higher mortality rate. This was found despite the fact that the case-fatality ratio in Africa for COVID-19 was reported to be lower than on other continents [13]. Our data might indicate that people admitted with COVID-19 tended to be sicker than their non-African counterparts.

Overall, the African cohort had a higher prevalence of AKI (12.7%) compared to the non-African cohort (7.2%). We showed that AKI was highly associated with overall mortality in the African cohort, and that those who had died were 41.7 times as likely to have had AKI compared to survivors. This strong link between AKI and mortality was much weaker in the non-African cohort. The adjusted odds ratio also showed that AKI was a very strong predictor of major adverse cardiovascular events, with an odds ratio of 660 in the African cohort. This strong association was again not reflected in the non-African cohort. Very few studies exist on prevalence of AKI in the African setting versus the global population, and those that have been conducted were small in scale. A systematic review and meta-analysis of chronic kidney disease in sub-Saharan Africa concluded from multiple studies that CKD prevalence was higher, with a reported 15.8 percent prevalence in the African population compared to the global burden of CKD, which stood at 10 percent in [14]. This may suggest a higher burden of renal injury already present in the African population in general. A recent review of the heart diseases in the tropics showed that a genetic mutation protecting individuals from African Trypanosomiasis led to higher rates of kidney disease in the same population [15]. Another explanation may be that patients admitted for COVID-19 in the African setting were generally presenting to healthcare facilities at a more advanced stage of disease. AKI should, however, be seen as a major warning sign and risk factor for mortality and MACE.

T wave changes allude to an abnormality in ventricular repolarisation and have been associated with poorer cardiovascular outcomes [17]. In the African population admitted for COVID-19, T wave changes were more prevalent than in the non-African cohort. A difference in QTc length was also observed when looking at the ECGs between the two cohorts. The risk of adverse cardiovascular events and sudden cardiac deaths were higher in a QTc interval >450 ms in men and >470 ms in women. A study done on abnormal QTc prolongation, as described above, reported that this was an independent predictor of sudden cardiac death and that those with QTc prolongation were at a threefold increase in risk of sudden cardiac death [16]. In the African cohort, ECG findings showed that the population had a significantly longer QTc ratio of 437 ms compared to the global population with 413 ms. This signifies the higher risk of sudden cardiac death. The T wave changes and QTc prolongation could suggest that African patients tended to be admitted with more severe disease.

## Strengths and Limitations

A major strength of the present study was our access to complete medical records of all hospital-screened RT-PCR-diagnosed COVID-19 patients. This allowed us to investigate the importance of pre-existing CVD comorbidities on the absolute risk of severe COVID-19 outcomes in an African Cohort and compare this to a non-African cohort.

There are, however, some limitations to our study. As we conducted a sub-analysis of the global study, our sample size was reduced and, therefore, so was the power of our analysis. We were only able to include nine African sites from six African countries and could therefore not make definitive inferences about the African population as a whole. The differences seen between the non-African and African cohorts may not be generalisable to all African countries. Factors that might influence generalizability of our study to the rest of Africa might be variations in patient demographics, healthcare systems, cultural practices, or differences in COVID-19 strains at the time of enrolment.

As by nature of observational study designs, the results prevented us from making definite inferences regarding causality. Given the diverse patient population and ethnic groups included, the p-values, particularly those assessing univariable and bivariable associations, should be interpreted cautiously. In this study, we only recruited hospitalized patients, who were more likely to present with more severe disease.

## Conclusion

Although other COVID-19 studies showed that the mortality rate of COVID-19 in Africa was significantly lower than in most WHO regions, this study found a higher overall mortality in hospitalised African patients [18]. Findings such as higher rates of AKI and risks for sudden cardiac death suggest that patients in Africa were admitted with more severe COVID-19 disease that might have influenced a higher in-hospital mortality rate, contributing to the higher overall mortality observed.
